# Perceiving visual negative stimuli in schizophrenia and bipolar disorder: Meta-analytic evidence of a common altered thalamic-parahippocampal-basal ganglia circuit

**DOI:** 10.1016/j.ynirp.2023.100173

**Published:** 2023-05-08

**Authors:** Alessandro Grecucci, Chiara Orsini, Gaia Lapomarda, Sara Sorella, Irene Messina

**Affiliations:** a Department of Psychology and Cognitive Sciences, University of Trento, Rovereto, Italy; b Center for Medical Sciences – CISMed, University of Trento, Trento, Italy; c Institute of Psychology, University of Innsbruck, Innsbruck, Austria; d Perception and Active Cognition Lab, Division of Science, New York University Abu Dhabi, United Arab Emirates; e Universitas Mercatorum, Rome, Italy

**Keywords:** Schizophrenia, Bipolar, Emotion, Meta-analysis, Perception

## Abstract

Despite the kraepelinian differentiation of schizophrenia and bipolar disorder, several data questioned this net subdivision and suggested a continuity between the two. An *expanded continuum hypothesis* was suggested, assuming a common psychotic core between the two disorders, as well as cognitive and affective differences. The present study aimed to investigate similarities and differences between schizophrenia and bipolar disorder for what entails the affective dimension of the *continuum*. A coordinate-based meta-analytic approach on neuroimaging data was applied to understand differences and similarities in the visual perception of negative stimuli in the two groups. The activation likelihood estimation analysis included 41 experiments on schizophrenia (schizophrenia versus healthy controls) and 27 experiments on bipolar disorder (bipolar versus healthy controls). Our conjunction analysis results revealed the presence of shared functional abnormalities in thalamic, parahippocampal, and basal ganglia areas, suggesting that these patients share an altered circuit responsible for a heightened elaboration of negative emotional stimuli. The subtraction analysis highlighted that the two groups present differences too. Schizophrenia patients show widespread abnormalities in limbic, temporal, sub-lobar and midbrain regions possibly involved in emotional processing and hallucinations. On the other hand, bipolar patients show alterations in frontal areas associated with emotional appraisal, regulation, and response inhibition. This study sheds light on both similarities and differences in the emotional processing of schizophrenic and bipolar patients, and may help to better characterise the affective features of these two conditions along a continuum.

## Highlights

• This study tested the SCZ-BD continuum hypothesis during negative emotion processing.
• Patients share hyperactivation in thalamus, basal ganglia and parahippocampal gyrus.
• SCZ differs from BD in limbic, temporal, claustral, midbrain areas.
• BD differs from SCZ in IFG, MFG and precentral gyrus.

## Graphical abstract

**Figure:**
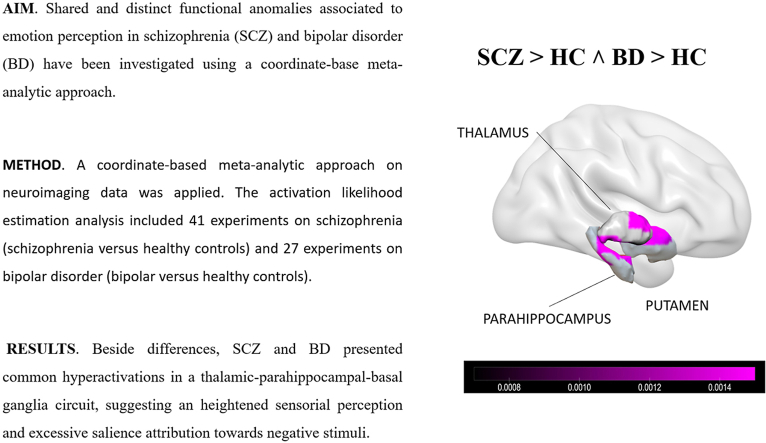

## 1 Introduction

Schizophrenia (SCZ) and bipolar disorder (BD) are two severe psychiatric illnesses whose diagnostic history intertwined over the course of time (see Angst, 2002 for a review). Despite they are still considered as separate disorders (DSM-V, American Psychiatric Association [APA], 2013; ICD-10, World Health Organization [WHO], 1993), similarities in the phenomenology of some symptoms (e.g., hallucinations, delusions), and increasing evidence coming from genetic, behavioural and neuroimaging research led several authors to consider these two conditions as part of a *continuum* (Crow, 1986; Sorella et al., 2019; for a review see Möller, 2003). There is evidence that first-degree relatives of either SCZ or BD patients are at increased risk to develop both disorders (Lichtenstein et al., 2009; Van Snellenberg and de Candia, 2009). In addition, genetic overlap between these disorders has been observed (Ahmadi et al., 2018; Badner and Gershon, 2002; Cheon et al., 2022; Vazza et al., 2007; for a review see Craddock et al., 2005), and, consistently, behavioural studies reported shared impairments in executive functions (Ancín et al., 2013; Kravariti et al., 2005), memory (Au et al., 2017; Pradhan et al., 2008), verbal fluency, motor speed (Caletti et al., 2013; Kuswanto et al., 2013), and emotion recognition (Goghari and Sponheim, 2013).

Brain imaging research provided relevant insights as well, looking for shared structural brain abnormalities between SCZ and BD (Cheon et al., 2022; Ellison-Wright and Bullmore, 2010; Hulshoff Pol et al., 2012; Knöchel et al., 2016; Yu et al., 2010; Zhao et al., 2021). Notably, Sorella et al. (2019) applied an unsupervised machine learning approach to investigate the overlap between SCZ and BD, by taking into account morphometric features as well as cognitive, affective, and symptomatology measures, and suggested the hypothesis of an *expanded continuum (*Sorella et al., 2019). According to this hypothesis, the similarities between SCZ and BD patients include different alterations in three cores: (a) the psychotic core, which involves portions of the medial parietal and temporo-occipital areas, parts of the cerebellum and the middle frontal gyrus; (b) the cognitive core, that is more compromised in SCZ rather than in BD and includes alterations in executive fronto-parietal areas; and, (c) the affective core, more altered in BD compared to SCZ, which includes portions of the temporal and occipital lobes, cerebellum, and frontal gyrus. These results are in line with those showing that cognitive impairments in BD patients are milder than the ones of SCZ patients (Bora and Pantelis, 2015; Krabbendam et al., 2005; Kuswanto et al., 2016; Sheffield et al., 2018), whereas the differences in the affective core are currently less understood. In the present study, we aimed to better characterise the affective dimension of the *expanded continuum hypothesis*, capitalising on a meta-analytic approach of brain functional studies on emotional processing in SCZ and BD patients.

It is well known that negative emotions can trigger hallucinations (Laloyaux et al., 2019; Waters et al., 2012) and delusions (for reviews see Freeman, 2007; Freeman and Garety, 2003), in both disorders. Importantly, emotions can also contribute to the maintenance of both delusions and hallucinations (Freeman and Garety, 2003). Emotion regulation deficits and emotional lability depending on fronto-limbic alterations are largely documented in BD (see the review by Townsend and Altshuler, 2012), while SCZ patients present dysfunctions in the domains of experience, perception, recognition, and expression of emotions (see Aleman and Kahn, 2005; Kohler and Martin, 2006; Trémeau, 2006 for reviews) and abnormalities in emotional memory (Herbener, 2008), in particular the intrusive and the negative ones – that can influence the content and meaning of hallucinations (Laloyaux et al., 2019; Waters et al., 2012). Previous meta-analyses on emotional processing in SCZ and BD separately showed BOLD signal alterations in limbic regions associated with emotional processing, such as the amygdala, anterior cingulated cortex, thalamic, occipital, frontal, and temporal areas (Chen et al., 2011; Dong et al., 2018; Houenou et al., 2011; H. J. Li et al., 2010; Taylor et al., 2012; Wegbreit et al., 2014). Another meta-analysis directly compared SCZ and BD patients, focusing on the processing of facial expressions (Delvecchio et al., 2013). Results showed different activity at the level of thalamus and cuneus, but any similarity emerged (Delvecchio et al., 2013). Taking into account the literature reviewed, no previous meta-analyses investigated shared and distinct brain functional mechanisms underlying the perception of visual negative stimuli (not limited to facial expressions) in both SCZ and BD. Thus, in the present study, we applied the activation likelihood estimation (ALE) meta-analytical technique on studies exploring the visual perception of a broad range of negative stimuli in SCZ and BD patients. The meta-analytic approach is a useful method to generalise the results of different studies on the same research topic (Gurevitch et al., 2018), reducing inconsistencies and discrepancies between them (Stone and Rosopa, 2017), and overcoming sample-size limitations (Berman and Parker, 2002). Furthermore, by computing the conjunction between two classes of patients, we can shed light on possible overlapping mechanisms and commonalities between disorders.

In light of what has emerged so far, we expect to find shared altered activity in regions underlying the abnormal affective experience both disorders suffer from (Chen et al., 2011; Houenou et al., 2011; Lapomarda et al., 2021a, 2021b; Dong et al., 2018; Taylor et al., 2012). Specifically, we hypothesise common alterations in a circuit including the thalamus and the basal ganglia, known to be involved in exaggerated affective experiences (e.g. emotions, impulses, mood, salience attribution) that characterise both disorders (Delvecchio et al., 2012; Howes and Kapur, 2009; Lapomarda et al., 2021a, 2021b; Sorella et al., 2019). We also expect common parahippocampal alterations due to its role in the elaboration of negative stimuli (for a review see Lew and Semendeferi, 2017) and in psychosis (Bodnar et al., 2011; Diederen et al., 2010; T. Yu et al., 2020). Furthermore, we predict abnormal activation in frontal areas involved in emotion control and regulation in BD (see Hou et al., 2017; and Townsend and Altshuler, 2012 for reviews, and Sorella et al., 2019), and in medial-temporal regions such as amygdala and hippocampus in SCZ for their role in emotional processes and dysfunctions associated with the pathology (Aleman and Kahn, 2005; Bolton et al., 2012; Bowen et al., 2018; Ganzola et al., 2014).

## 2 Materials and methods

### 2.1 Search criteria and study selection

Studies were collected through two systematic reviews (one for each clinical group) conducted in July 2020 from three online databases: PubMed (https://www.ncbi.nlm.nih.gov/pubmed/), Web of Science (http://www.webofknowledge.com/) and ScienceDirect (https://www.sciencedirect.com). The research queries for the schizophrenia group were *schizophrenia AND (fMRI OR “functional magnetic resonance”) AND emotion AND (processing OR perception) AND (face OR faces OR facial)* for facial stimuli and *schizophrenia AND (fMRI OR “functional magnetic resonance”) AND (IAPS OR “international affective picture system”)* for the IAPS ones. Similarly, the search terms for the bipolar group were *bipolar AND (fMRI OR “functional magnetic resonance”) AND emotion AND (processing OR perception) AND (face OR faces OR facial)* for facial stimuli and *bipolar AND (fMRI OR “functional magnetic resonance”) AND (IAPS OR “international affective picture system”)* for IAPS stimuli.

This research yielded a total of 4238 results for schizophrenia and 2961 results for bipolar disorder, which have been consequently selected according to the following selection criteria.

a) Studies published in English.
b) Patients specifically diagnosed with schizophrenia (schizophrenia spectrum disorders were excluded) or bipolar disorder (Type I and II) and control group composed of healthy participants.
c) Participants entered at least in their eighteenth year of age (17 years old) considering the standard deviation range reported in papers' demographical section.
d) Use of fMRI in order to evaluate differences in emotion perception activation between pathological and healthy groups.
e) The task used negative IAPS (Lang et al., 2008) or facial emotion (angry, sad, fearful, disgusted) stimuli and contrasted them, respectively, with neutral IAPS or neutral faces in the same experimental condition (e.g. 2-back negative vs. 1-back neutral experiments were excluded, while the 2-back negative vs. 2-back neutral ones were included).
f) fMRI ROIs or whole brain coordinates reported and provided in MNI or Talairach stereotactic spaces. When studies presented both ROIs and whole brain data, all of them were included.

Through this study selection, 20 studies for the schizophrenia group and 17 for the bipolar group were identified. Aggregated results were used if available. When not aggregated (e.g. there were only separate analyses and results for different patients subgroups, different emotions, tasks, or different emotional stimuli intensity/arousal/consciousness), these data were considered as separated results. These experiments were then inserted separately in our datasets and analyses. In line with that, 41 experiments for the schizophrenia group and 27 experiments for the bipolar disorder group were identified (Table 1 and Supplementary Table S1). Participants were 2548, specifically 840 SCZ (mean age = 31.08 ± 8.51), 392 BD (mean age = 36.30 ± 10.83) and 1316 HC (mean age = 31.78 ± 9.27).

**Table 1:** Main details and description of the studies included in the meta-analysis.

N° paper	Studies	N	Age	Stimuli	Task	Contrast	WB/ROIs	Foci hypoactivations	Foci hyperactivations
SCHIZOPHRENIA									
1	Hall et al. (2008)	19 (SCZ)24 (HC)	37.7 ± 8.4 (SCZ)35.1 ± 9.7 (HC)	FACES	Gender ID	FEAR vs NEUTRAL	WB + ROIs	3	–
2	H. J. Li et al. (2012)	12 (SCZ)12 (HC)	29.8 ± 9.24 (SCZ)29.25 ± 7.24 (HC)	FACES	Judge valence	FEAR vs NEUTRAL	WB + ROIs	1	1
3	Michalopoulou et al. (2008)	11 (SCZ)9 (HC)	35 ± 9 (SCZ)32 ± 6 (HC)	FACES	Gender ID	FEAR vs NEUTRAL	WB	6	–
4	Mier et al. (2014)	11 (SCZ)16 (HC)	32.45 ± 7.66 (SCZ)34.5 ± 6.47 (HC)	FACES	Emotion ID	FEAR vs NEUTRAL	ROIs	2	–
5		11 (SCZ)16 (HC)	32.45 ± 7.66 (SCZ)34.5 ± 6.47 (HC)	FACES	Emotion ID	ANGER vs NEUTRAL	ROIs	1	–
6		11 (SCZ)16 (HC)	32.45 ± 7.66 (SCZ)34.5 ± 6.47 (HC)	FACES	Emotion ID	DISGUST vs NEUTRAL	ROIs	–	–
7	Williams et al. (2004)	27 (SCZ paranoid + non-paranoid)22 (HC)	27.3 ± 9.6 (SCZ paranoid + non-paranoid)27.2 ± 8.1 (HC)	FACES	Gender ID	FEAR vs NEUTRAL	WB	5	–
8	Williams et al. (2007)	13 (SCZ paranoid)13 (HC)	26.9 ± 9.1 (SCZ paranoid)25.1 ± 8.1 (HC)	FACES	Gender ID	With arousal FEAR vs NEUTRAL	WB + ROIs	2	–
9		14 (SCZ non-paranoid)13 (HC)	27.8 ± 10.4 (SCZ non-paranoid)25.1 ± 8.1 (HC)	FACES	Gender ID	With arousal FEAR vs NEUTRAL	WB + ROIs	2	–
10		13 (SCZ paranoid)13 (HC)	26.9 ± 9.1 (SCZ paranoid)25.1 ± 8.1 (HC)	FACES	Gender ID	Without arousal FEAR vs NEUTRAL	WB + ROIs	1	–
11		14 (SCZ non-paranoid)13 (HC)	27.8 ± 10.4 (SCZ non-paranoid)25.1 ± 8.1 (HC)	FACES	Gender ID	Without arousal FEAR vs NEUTRAL	WB + ROIs	1	–
12		13 (SCZ paranoid)13 (HC)	26.9 ± 9.1 (SCZ paranoid)25.1 ± 8.1 (HC)	FACES	Gender ID	With arousal ANGER vs NEUTRAL	WB + ROIs	1	–
13		14 (SCZ non-paranoid)13 (HC)	27.8 ± 10.4 (SCZ non-paranoid)25.1 ± 8.1 (HC)	FACES	Gender ID	With arousal ANGER vs NEUTRAL	WB + ROIs	–	–
14		13 (SCZ paranoid)13 (HC)	26.9 ± 9.1 (SCZ paranoid)25.1 ± 8.1 (HC)	FACES	Gender ID	Without arousal ANGER vs NEUTRAL	WB + ROIs	2	–
15		14 (SCZ non-paranoid)13 (HC)	27.8 ± 10.4 (SCZ non-paranoid)25.1 ± 8.1 (HC)	FACES	Gender ID	Without arousal ANGER vs NEUTRAL	WB + ROIs	2	–
16		13 (SCZ paranoid)13 (HC)	26.9 ± 9.1 (SCZ paranoid)25.1 ± 8.1 (HC)	FACES	Gender ID	With arousal DISGUST vs NEUTRAL	WB + ROIs	1	–
17		14 (SCZ non-paranoid)13 (HC)	27.8 ± 10.4 (SCZ non-paranoid)25.1 ± 8.1 (HC)	FACES	Gender ID	With arousal DISGUST vs NEUTRAL	WB + ROIs	1	–
18		13 (SCZ paranoid)13 (HC)	26.9 ± 9.1 (SCZ paranoid)25.1 ± 8.1 (HC)	FACES	Gender ID	Without arousal DISGUST vs NEUTRAL	WB + ROIs	–	–
19		14 (SCZ non-paranoid)13 (HC)	27.8 ± 10.4 (SCZ non-paranoid)25.1 ± 8.1 (HC)	FACES	Gender ID	Without arousal DISGUST vs NEUTRAL	WB + ROIs	1	–
20	Surguladze et al. (2011)	16 (SCZ conventional antipsychotic)16 (HC)	43.7 ± 9.4 (SCZ conventional antipsychotic)40.4 ± 12.8 (HC)	FACES	Gender ID	FEAR vs NEUTRAL	WB + ROIs	1	1
21		16 (SCZ risperidone)16 (HC)	42.6 ± 11.7 (SCZ risperidone)40.4 ± 12.8 (HC)	FACES	Gender ID	FEAR vs NEUTRAL	WB + ROIs	–	–
22	Lindner et al. (2014)	36 (SCZ)40 (HC)	30.8 ± 7.9 (SCZ)29.5 ± 8.3 (HC)	FACES	Passive view	Masked DISGUST vs NEUTRAL	WB + ROIs	4	2
23		36 (SCZ)40 (HC)	30.8 ± 7.9 (SCZ)29.5 ± 8.3 (HC)	FACES	Passive view	Unmasked DISGUST vs NEUTRAL	WB + ROIs	1	8
24	Rauch et al. (2010)	12 (SCZ)12 (HC)	27.7 ± 7.5 (SCZ)26.9 ± 6.1 (HC)	FACES	Rate valence (masked faces)	Masked SAD vs NEUTRAL	ROIs	–	3
25	Lindner et al. (2016)	36 (SCZ with flat affect + without)40 (HC)	30.6 ± 8 (SCZ with flat affect + without)29.5 ± 8.3 (HC)	FACES	View and memorise	Masked FEAR vs NEUTRAL	WB + ROIs	–	–
26		36 (SCZ with flat affect + without)40 (HC)	30.6 ± 8 (SCZ with flat affect + without)29.5 ± 8.3 (HC)	FACES	View and memorise	Masked DISGUST vs NEUTRAL	ROIs	–	–
27		34 (SCZ with flat affect + without)40 (HC)	30.6 ± 8 (SCZ with flat affect + without)29.5 ± 8.3 (HC)	FACES	View and memorise	Unmasked FEAR vs NEUTRAL	WB + ROIs	–	5
28		34 (SCZ with flat affect + without)40 (HC)	30.6 ± 8 (SCZ with flat affect + without)29.5 ± 8.3 (HC)	FACES	View and memorise	Unmasked DISGUST vs NEUTRAL	ROIs	–	2
29	Das et al. (2007)	14 (first episode SCZ)14 (HC)	20.4 ± 3.3 (first episode SCZ)23.1 ± 5.9 (HC)	FACES	Emotion ID	Unmasked FEAR vs NEUTRAL	ROIs	6	5
30		13 (first episode SCZ)14 (HC)	20.4 ± 3.3 (first episode SCZ)23.1 ± 5.9 (HC)	FACES	Emotion ID	Masked FEAR vs NEUTRAL	ROIs	5	3
31	Becerril and Barch (2011)	38 (SCZ)32 (HC)	36.66 ± 9.12 (SCZ)36.19 ± 10.86 (HC)	FACES	2-back working memory	FEAR vs NEUTRAL	WB + ROIs	–	18
32	Reske et al. (2009)	18 (first episode SCZ)18 (HC)	31.94 ± 6.41 (first episode SCZ)31.94 ± 6.03 (HC)	FACES	Emotion ID	SAD vs NEUTRAL	WB	–	1
33	Takahashi et al. (2004)	15 (SCZ)15 (HC)	29.0 ± 6.9 (SCZ)29.1 ± 7.8 (HC)	IAPS	Categorise the emotion felt	NEGATIVE vs NEUTRAL	WB	10	–
34	Mendrek et al. (2012)	17 (SCZ follicular phase)15 (HC)	32.86 ± 6.56 (SCZ follicular phase)29.28 ± 9.27 (HC)	IAPS	Passive view + indicate images with a person/part of it	NEGATIVE vs NEUTRAL	WB + ROIs	–	–
35		17 (SCZ luteal phase)15 (HC)	32.86 ± 6.56 (SCZ luteal phase)29.28 ± 9.27 (HC)	IAPS	Passive view + indicate images with a person/part of it	NEGATIVE vs NEUTRAL	WB + ROIs	15	1
36	Champagne et al. (2012)	43 (SCZ female + male)43 (HC)	32.86 ± 6.56 (SCZ female)31.36 ± 7.36 (SCZ male)29.28 ± 9.27 (HC female)30.55 ± 7.81 (HC male)	IAPS	Passive view	NEGATIVE vs NEUTRAL	WB	–	–
37	Hägele et al. (2016)	37 (SCZ)40 (HC)	31 ± 9.4 (SCZ)36.3 ± 11.8 (HC)	IAPS	Passive view + confirm picture view	NEGATIVE vs NEUTRAL	WB + ROIs	–	–
38	Lakis et al. (2011)	37 (SCZ)37 (HC)	32.46 ± 7.66 (SCZ)31.81 ± 6.91 (HC)	IAPS	Episodic memory retrieval (yes/no recognition paradigms)	Low arousal NEGATIVE vs NEUTRAL	WB	3	–
39		37 (SCZ)37 (HC)	32.46 ± 7.66 (SCZ)31.81 ± 6.91 (HC)	IAPS	Episodic memory retrieval (yes/no recognition paradigms)	High arousal NEGATIVE vs NEUTRAL	WB	2	–
40	Anticevic et al. (2011)	23 (SCZ)23 (HC)	36.39 ± 9.54 (SCZ)37.18 ± 7.59 (HC)	IAPS	Delayed match-to-sample visual working memory (IAPS = distractors)	NEGATIVE vs NEUTRAL	WB + ROIs	2	–
41	Diaz et al. (2011)	11 (SCZ)17 (HC)	32.57 ± 12.7 (SCZ)24.01 ± 3.89 (HC)	IAPS	View IAPS during maintaining memoranda in verbal working memory task (IAPS = distractors)	NEGATIVE vs NEUTRAL	WB + ROIs	5	–
BIPOLAR DISORDER									
1	Lawrence et al. (2004)	12 (BD-I euthymic)11 (HC)	41 ± 11 (BD-I euthymic)41 ± 11 (HC)	FACES	Gender ID	50% FEAR vs NEUTRAL	ROIs	2	1
2		12 (BD-I euthymic)11 (HC)	41 ± 11 (BD-I euthymic)41 ± 11 (HC)	FACES	Gender ID	100% FEAR vs NEUTRAL	ROIs	–	1
3		12 (BD-I euthymic)11 (HC)	41 ± 11 (BD-I euthymic)41 ± 11 (HC)	FACES	Gender ID	50% SAD vs NEUTRAL	ROIs	3	2
4		12 (BD-I euthymic)11 (HC)	41 ± 11 (BD-I euthymic)41 ± 11 (HC)	FACES	Gender ID	100% SAD vs NEUTRAL	ROIs	2	2
5	Lennox et al. (2004)	10 (BD-I manic)12 (HC)	37.3 ± 12.8 (BD-I manic)32.6 ± 10.7 (HC)	FACES	Rate Emotion Intensity	SAD vs NEUTRAL	WB + ROIs	7	5
6	Malhi et al. (2007)	10 (BD-I euthymic)10 (HC)	33.5 ± 8.7 (BD-I euthymic)32.4 ± 6.4 (HC)	FACES	Emotion ID	FEAR vs NEUTRAL	WB	1	9
7		10 (BD-I euthymic)10 (HC)	33.5 ± 8.7 (BD-I euthymic)32.4 ± 6.4 (HC)	FACES	Emotion ID	DISGUST vs NEUTRAL	WB	15	4
8	Jogia et al. (2008)	8 (BD-I baseline)12 (HC)	42.1 ± 11.8 (BD-I baseline)41.8 ± 10.9 (HC)	FACES	Emotion ID	SAD vs NEUTRAL	WB	5	1
9	Sagar et al. (2013)	23 (BD-I euthymic)18 (HC)	26.65 ± 6.65 (BD-I euthymic)23.11 ± 3.15 (HC)	FACES	Backward-masked affect paradigm (Gender ID)	Masked FEAR vs NEUTRAL	ROIs	4	22
10	Marchand et al. (2011)	16 (BD-II depressed)19 (HC)	32.9 ± 7.5 (BD-II depressed)33.7 ± 12.5 (HC)	FACES	Match Identity AND Expression	FEAR vs NEUTRAL	WB + ROIs	–	–
11	Surguladze et al. (2010)	20 (BD-I euthymic)20 (HC)	42.7 ± 10.4 (BD-I euthymic)41.9 ± 11.6 (HC)	FACES	Gender ID	FEAR vs NEUTRAL	WB + ROIs	–	2
12	Mullin et al. (2012)	22 (BD-I euthymic)19 (HC)	31.68 ± 8.96 (BD-I euthymic)32.54 ± 6.56 (HC)	FACES	2-back working memory	FEAR vs NEUTRAL	ROIs	–	5
13	Chen et al. (2006)	8 (BD-I manic)8 (HC)	39 ± 13.44 (BD-I manic)38.75 ± 12.5 (HC)	FACES	Rate emotion intensity + Rate colour intensity	FEAR vs NEUTRAL	WB	–	8
14		8 (BD-I manic)8 (HC)	39 ± 13.44 (BD-I manic)38.75 ± 12.5 (HC)	FACES	Rate emotion intensity + Rate colour intensity	SAD vs NEUTRAL	WB	–	1
15		8 (BD-I depressed)8 (HC)	41.88 ± 12.09 (BD-I depressed)38.75 ± 12.5 (HC)	FACES	Rate emotion intensity + Rate colour intensity	FEAR vs NEUTRAL	WB	–	8
16		8 (BD-I depressed)8 (HC)	41.88 ± 12.09 (BD-I depressed)38.75 ± 12.5 (HC)	FACES	Rate emotion intensity + Rate colour intensity	SAD vs NEUTRAL	WB	–	–
17	Grotegerd et al. (2014)	22 (BD-I depressed)22 (HC)	42 ± 11 (BD-I depressed)41.1 ± 10.9 (HC)	FACES	Rate masked valence	Masked SAD vs NEUTRAL	ROIs	1	–
18	Deveney et al. (2014)	22 (BD-I/II adults, euthymic or depressed)19 (HC adults)	35.54 ± 11.2 (BD-I/II adults, euthymic or depressed)32.8 ± 11.4 (HC adults)	FACES	Rate hostility (explicit)	ANGER vs NEUTRAL	WB + ROIs	1	–
19		22 (BD-I/II adults, euthymic or depressed)19 (HC adults)	35.54 ± 11.2 (BD-I/II adults, euthymic or depressed)32.8 ± 11.4 (HC adults)	FACES	Rate nose width (implicit)	ANGER vs NEUTRAL	WB + ROIs	–	–
20	Rootes-Murdy et al. (2019)	8 (BD-I lithium responders, euthymic)21 (HC)	41.63 ± 14.03 (BD-I lithium responders, euthymic)36.33 ± 12.96 (HC)	FACES	Emotion induction (view)	FEAR vs NEUTRAL	WB	1	–
21		4 (BD-I lithium non-responders, euthymic)21 (HC)	29.75 ± 12.18 (BD-I lithium non-responders, euthymic)36.33 ± 12.96 (HC)	FACES	Emotion induction (view)	FEAR vs NEUTRAL	WB	–	–
22	Bermpohl et al. (2009)	10 (BD-I manic)10 (HC)	37.9 ± 13.2 (BD-I manic)35.8 ± 12.9 (HC)	IAPS	Passive view + confirm picture view	NEGATIVE vs NEUTRAL	ROIs	–	–
23	Cerullo et al. (2014)	25 (BD-I depressed)25 (HC)	30 ± 8 (BD-I depressed)26 ± 7 (HC)	IAPS	Continuous performance task with emotional and neutral distracters (CPT-END)	NEGATIVE vs NEUTRAL	WB	5	11
24	L. Li et al. (2019)	13 (BD-I lithium, euthymic)16 (HC)	29.6 ± 9.12 (BD-I lithium, euthymic)29.4 ± 7.47 (HC)	IAPS	Rate valence	NEGATIVE vs NEUTRAL	WB + ROIs	–	–
25		16 (BD-I valproate, euthymic)16 (HC)	32.8 ± 8.18 (BD-I valproate, euthymic)29.4 ± 7.47 (HC)	IAPS	Rate valence	NEGATIVE vs NEUTRAL	WB + ROIs	–	–
26	Hägele et al. (2016)	12 (BD-I manic)40 (HC)	40.7 ± 14.4 (BD-I manic)36.3 ± 11.8 (HC)	IAPS	Passive view + confirm picture view	NEGATIVE vs NEUTRAL	WB + ROIs	–	–
27	Ellard et al. (2019)	39 (BD-I depressed)36 (HC)	36.69 ± 12.92 (BD-I depressed)34.69 ± 12.64 (HC)	IAPS	Multi-Source Interference Task (MSIT)	NEGATIVE vs NEUTRAL	ROIs	–	–

### 2.2 Activation likelihood estimation procedure

This neuroimaging coordinate-based meta-analysis was performed using the activation likelihood estimation (ALE) procedure (Eickhoff et al., 2009, 2012; Turkeltaub et al., 2012), through the GingerALE software v3.0.2 (http://brainmap.org/). The ALE algorithm estimates the significant convergence between neuroimaging experiments (Eickhoff et al., 2012). It models different studies’ foci as three-dimensional Gaussian probability distributions centered at the foci coordinates, therefore considering the spatial uncertainty associated to the determination of their xyz locations (Eickhoff et al., 2009; Turkeltaub et al., 2002). Then the maximum probability relative to each Gaussian-modeled foci belonging to the same experiment is calculated, obtaining Modeled Activation (MA) maps: the voxelwise union of the latter provides the ALE map (Turkeltaub et al., 2012). A permutation test discriminates the effective significant convergence from the noise (Eickhoff et al., 2009, 2012).

In the present study, the selected foci reported in Talairach space (Talairach and Tournoux, 1988) have been converted in the Montreal Neurological Institute (MNI) one (Collins et al., 1994; Evans et al., 1993) through the GingerALE “Convert Foci” tool, which performs the transformation using the icbm2tal algorithm developed by Lancaster et al., in 2007. Again, when the selected studies applied the mni2tal transformation (Brett et al., 2001, 2002), its effect has been removed using this tool in order to increase the precision of our study (Laird et al., 2010). After this spatial conversion, two separate datasets (one for the hyperactivation and one for the hypoactivation) for each clinical group were created, generating a total of four files (SCZ > HC = 41 experiments, 50 foci, 1715 participants; SCZ < HC = 41 experiments, 86 foci, 1715 participants; BD > HC = 27 experiments, 82 foci, 833 participants; BD < HC = 27 experiments, 47 foci, 833 participants). Before starting the analyses, GingerALE preference “Cluster Analysis Labels” was set on “Gray Matter Only”. Then, the previously mentioned files were separately entered in GingerALE as inputs for single datasets analyses (first-level analyses), applying a cluster-level family-wise error (FWE) < 0.05, threshold permutations equal to 2000 and *p*-value <0.05.

Since the aim of this study was to directly compare brain activation abnormalities between SCZ and BD by contrast analyses, we launched the same procedure also for two BD and SCZ pooled datasets: one for patients hypoactivation (POOLED < HC = 68 experiments, 133 foci, 2548 participants) and one for hyperactivation (POOLED > HC = 68 experiments, 132 foci, 2548 participants). This first step provided above threshold ALE maps representing separately the hypoactivation and hyperactivation of bipolar disorder, schizophrenia, and pooled patients group with respect to healthy controls. Furthermore, in order to verify whether altered functional substrates presenting a difference in the direction of abnormal activation exist between the two clinical groups, two additional pooled datasets were created. Specifically, the BD > HC dataset was pooled with the SCZ < HC one (leading to a pooled file containing 68 experiments, 168 foci, and 2548 participants) and the file BD < HC was pooled with the SCZ > HC one (pooled file containing 68 experiments, 97 foci, and 2548 participants). These outputs were then used as inputs for the contrast (second-level) analyses (Eickhoff et al., 2011). Parameters for these computations were set at *p*-value <0.05, *p*-value permutations = 2000 and minimum cluster volume = 50 mm³. This procedure operates at two levels, elaborating both statistically significant differences and overlapping elements between the two ALE datasets. Differences are the product of the direct subtraction of the second-level analyses datasets (ALE images) of each group from the other. Instead, similarities are the results of the conjunction generated with the voxel-wise minimum value of these ALE datasets (Sorella et al., 2021). Brain plots were generated with SurfIce (https://www.nitrc.org/plugins/mwiki/index.php/surfice:MainPage).

## 3 Results

In the present section, we describe the results obtained in four contrasts of interest from second-level analyses: overlapping hyper-/hypo-activation and distinct hyper-/hypo-activation. The analyses carried out in order to look for the presence of common abnormal alterations presenting a difference in the direction of activation across clinical groups (BD > HC ∧ SCZ < HC and BD < HC ∧ SCZ > HC) gave no results, and will not be discussed further in this section. See Supplementary Tables S2 and S3 for the first level analysis data (SCZ versus HC and BD versus HC).

### 3.1 Common imparments in SCZ and BD

**Fig. 1:**
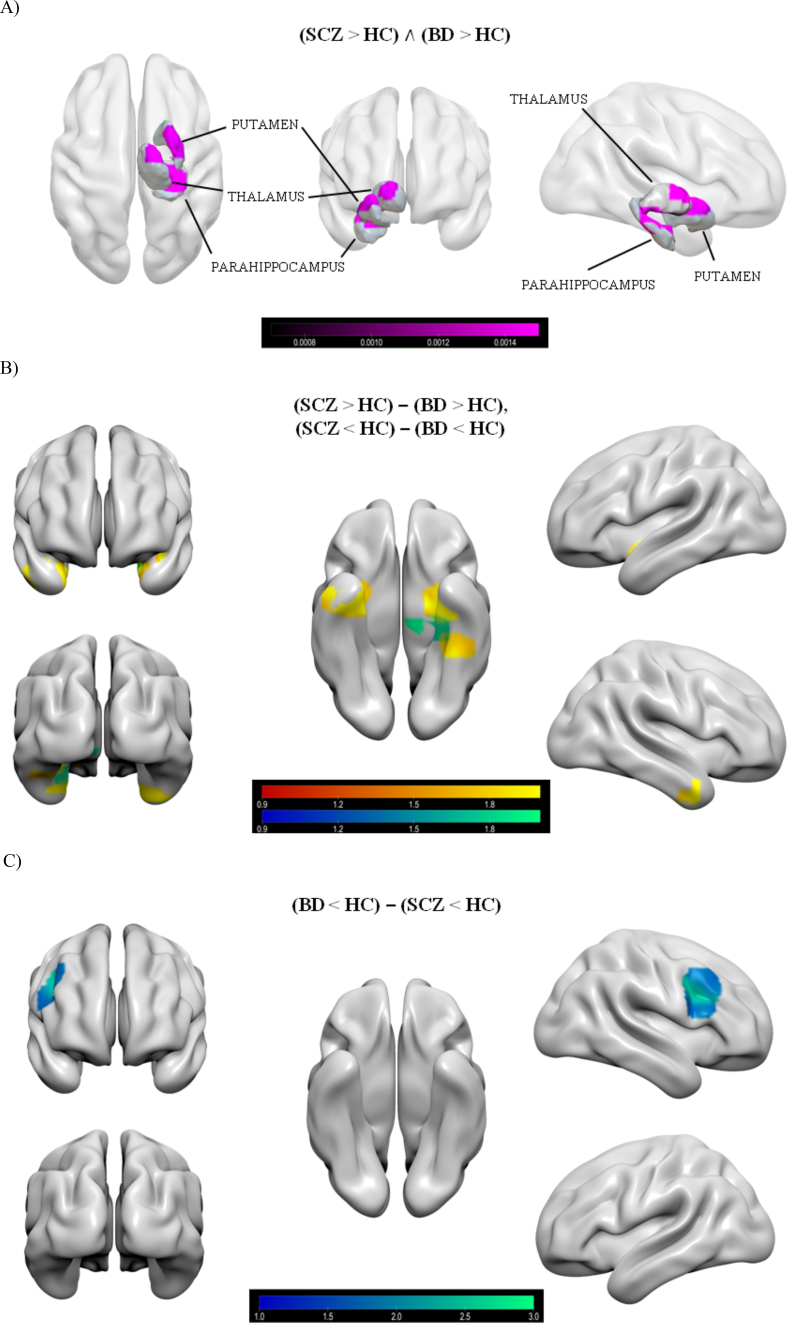
Brain functional abnormal activations in schizophrenia and bipolar patients. On the top (A), schizophrenics and bipolars common impairments (hyperactivations) are shown in magenta (ALE scores). In the middle (B), functional anomalies associated to schizophrenia (Z scores). In the bottom (C), functional anomalies associated to bipolar disorder (Z scores). In Fig. 1(B) and (C), hyperactivations are shown in the red-yellow gradient; hypoactivations are reported in the blue-green gradient. (For interpretation of the references to colour in this figure legend, the reader is referred to the Web version of this article.)

The conjunction analysis included 68 experiments and 2548 participants, yielding a total of 132 foci of increased brain activity and 133 foci of decreased brain activity in SCZ and BD patients compared to HC. This analysis revealed the presence of three right lateralised overlapping clusters of common increased brain activity in SCZ and BD patients compared to HC, located in sub-lobar and limbic areas. The first and larger cluster (984 mm³) extends entirely in right Sub-lobar areas, specifically in the Lentiform Nucleus (94.3% Putamen, 5.7% Lateral Globus Pallidus). The second cluster presents a smaller size (264 mm³) and is located in its entirety in the right Limbic Lobe, in the Parahippocampal Gyrus (54.5% Brodmann area 35, 45.5% Brodmann area 28). The third cluster (216 mm³) is situated in right Sub-lobar areas, specifically it extends in the Lentiform Nucleus (61.1%) and in the Thalamus (38.9%) (in particular: 27.8% Lateral Globus Pallidus, 27.8% Ventral Lateral Nucleus, 11.1% Medial Globus Pallidus, 5.6% Ventral Anterior Nucleus). No significant clusters of decreased brain activity emerged from this analysis. See Fig. 1 and Table 2.

**Table 2:** Significant clusters presenting shared abnormal activations in schizophrenia and bipolar disorder compared to healthy controls.

Cluster	Side	Label (Nearest Gray Matter within 5 mm)	BA	Peaks (MNI)			ALE Score
				x	y	z	
HYPERACTIVATIONS OVERLAP (SCZ > HC) ∧ (BD > HC)							
1	R	Sub-lobar, Lentiform Nucleus, Putamen		30	2	−6	0.009
2	R	Limbic Lobe, Parahippocampal Gyrus	35	28	−26	−18	0.003
2	R	Limbic Lobe, Parahippocampal Gyrus	35	24	−24	−18	0.003
3	R	Sub-lobar, Lentiform Nucleus		16	2	2	0.003
3	R	Sub-lobar, Thalamus, Ventral Lateral Nucleus		14	−8	2	0.003

### 3.2 SCZ versus BD

The subtraction analysis between SCZ and BD groups included: (a) 41 experiments for the contrast SCZ vs HC and 1715 participants, yielding 50 foci of increased brain activity and 86 foci of decreased brain activity; (b) 27 experiments for the contrast BD vs HC and 833 participants, yielding a total of 82 foci of increased brain activity and 47 foci of decreased brain activity. For these analyses, each cluster peaks' details are reported in Table 3. See Fig. 1.

**Table 3:** Significant clusters presenting different abnormal activations between schizophrenia and bipolar disorder compared to healthy controls.

Cluster	Side	Label (Nearest Gray Matter within 5 mm)	BA	Peaks (MNI)			*P*	*Z*
				x	y	z		
GREATER HYPERACTIVATION IN SCHIZOPHRENIA (SCZ > HC) – (BD > HC)								
1	L	Sub-lobar, Claustrum		−34	−8	−12	0.006	2.543
1	L	Limbic Lobe, Parahippocampal Gyrus, Hippocampus		−32	−12	−16	0.007	2.484
1	L	Limbic Lobe, Parahippocampal Gyrus, Hippocampus		−36	−10	−16	0.009	2.366
1	L	Limbic Lobe, Parahippocampal Gyrus, Hippocampus		−34	−12	−22	0.011	2.308
1	L	Limbic Lobe, Parahippocampal Gyrus, Amygdala		−31.1	−5.3	−16.2	0.014	2.197
1	L	Limbic Lobe, Parahippocampal Gyrus, Hippocampus		−28	−12	−27	0.015	2.183
1	L	Limbic Lobe, Parahippocampal Gyrus, Amygdala		−26	−12	−21	0.015	2.170
1	L	Limbic Lobe, Parahippocampal Gyrus, Amygdala		−22	−7	−18	0.016	2.144
1	L	Limbic Lobe, Parahippocampal Gyrus, Amygdala		−29.5	−1.5	−20	0.018	2.108
1	L	Limbic Lobe, Parahippocampal Gyrus, Amygdala		−28	−4.7	−24	0.025	1.969
1	L	Temporal Lobe, Superior Temporal Gyrus	38	−33	2	−19	0.038	1.780
1	L	Limbic Lobe, Parahippocampal Gyrus, Amygdala		−31.3	−2.1	−27.6	0.025	1.969
2	R	Limbic Lobe, Uncus, Amygdala		32.5	−6.6	−28.5	0.025	1.969
2	R	Limbic Lobe, Uncus, Amygdala		26.8	−2.8	−28.4	0.025	1.969
2	R	Limbic Lobe, Uncus	28	30	4	−26	0.023	1.995
2	R	Limbic Lobe, Uncus	28	36.6	−1	−31.3	0.025	1.969
2	R	Limbic Lobe, Parahippocampal Gyrus, Amygdala		26.7	−1.3	−23	0.042	1.734
3	L	Temporal Lobe, Fusiform Gyrus	20	−38.9	−37	−18.9	0.024	1.986
3	L	Limbic Lobe, Parahippocampal Gyrus	36	−35.3	−33	−20	0.040	1.757
3	L	Temporal Lobe, Fusiform Gyrus	36	−43	−36	−25	0.046	1.690
GREATER HYPOACTIVATION IN SCHIZOPHRENIA (SCZ < HC) – (BD < HC)								
1	L	Limbic Lobe, Parahippocampal Gyrus	28	−22	−22	−24	0.024	1.986
1	L	Limbic Lobe, Parahippocampal Gyrus	35	−20	−26	−26	0.025	1.960
1	L	Limbic Lobe, Parahippocampal Gyrus	35	−26	−22	−25	0.034	1.832
2	L	Brainstem, Midbrain, Substantia Nigra		−8	−22	−14	0.026	1.943
2	L	Brainstem, Midbrain, Red Nucleus		−8	−22	−10	0.03	1.881
GREATER HYPOACTIVATION IN BIPOLAR DISORDER (BD < HC) – (SCZ < HC)								
1	R	Frontal Lobe, Precentral Gyrus	6	38.7	10.2	28	0.007	2.484
1	R	Frontal Lobe, Middle Frontal Gyrus	9	46	18	26	0.025	1.960
1	R	Frontal Lobe, Inferior Frontal Gyrus	9	46	18	22	0.009	2.387
1	R	Frontal Lobe, Precentral Gyrus	9	45.3	12.9	29.3	0.01	2.326
1	R	No Gray Matter found		34.3	12	35	0.011	2.290
1	R	Frontal Lobe, Inferior Frontal Gyrus	9	48	16	18	0.029	1.896
1	R	Frontal Lobe, Precentral Gyrus	9	44	24	35	0.031	1.866
1	R	Frontal Lobe, Precentral Gyrus	9	39.2	13.5	34.2	0.007	2.484
1	R	Frontal Lobe, Inferior Frontal Gyrus	9	50	10	20	0.046	1.690
1	R	No Gray Matter found		28.8	7.8	35	0.048	1.670

This analysis revealed three clusters of increased brain activity which specifically characterise SCZ. The first cluster (3632 mm³) is located in the left Limbic Lobe: it includes the Parahippocampal Gyrus (87.9%) and the Uncus (12.1%) (in particular: 87.9% Amygdala, 9.9% Hippocampus, 2.1% Brodmann area 34). The second cluster (2560 mm³) includes the right Limbic Lobe (93.3%) and the right Temporal Lobe (6.7%). Specifically, it includes the Uncus (58.3%), the Parahippocampal Gyrus (35%), the Fusiform Gyrus (3.3%) and the Middle Temporal Gyrus (3.3%) (in particular: 58.3% Amygdala, 30% Brodmann area 28, 5% Brodmann area 34, 3.3% Brodmann area 20, 3.3% Brodmann area 21). The third and last cluster (608 mm³) is located in left Cerebrum (97.6%) and left Cerebellum (2.4%). It includes the Limbic Lobe (58.5%), the Temporal Lobe (39%) and the Anterior Lobe (2.4%). Specifically, it includes the Parahippocampal Gyrus (58.5%), the Fusiform Gyrus (39%) and the Culmen (2.4%) (in particular: 63.4% Brodmann area 36, 34.1% Brodmann area 20).

The subtraction analysis also revealed two clusters of decreased brain activity specific for SCZ. The first cluster (760 mm³) is located in the left Limbic Lobe, specifically in the Parahippocampal Gyrus (73.1% Brodmann area 35, 26.9% Brodmann area 28). The second cluster (64 mm³) includes the left Midbrain (100% Substantia Nigra).

Finally, a significant right lateralised cluster of decreased brain activity specific for BD was found. This cluster (4272 mm³) is located in the right Frontal Lobe. Specifically, it includes the Precentral Gyrus (66.7%), the Inferior Frontal Gyrus (18.3%), and the Middle Frontal Gyrus (15%) (in particular: 90% Brodmann area 9, 10% Brodmann area 6).

No significant clusters of increased brain activity which characterise BD were found.

## 4 Discussion

Despite the still in use dichotomous division of schizophrenia (SCZ) and bipolar disorder (BD), several findings questioned this position and led to think about the existence of a *continuum* between these conditions (Crow, 1986; Möller, 2003; Sorella et al., 2019). The present study focused on the affective dimension of the *expanded continuum hypothesis* (Sorella et al., 2019), and aimed to identify similarities and differences in this domain by using a coordinate-based meta-analytic approach of neuroimaging studies. Specifically, we focused on studies that investigated the perception of negative visual stimuli in SCZ and BD. Taking into consideration the literature reviewed, this is the first meta-analysis that directly compares abnormal brain activations of SCZ and BD patients during the visual perception of negative emotional stimuli.

### 4.1 Overlap between schizophrenia and bipolar disorder

We found shared abnormalities between SCZ and BD in brain functioning involved in the perception and elaboration of negative stimuli. Specifically, the conjunction analysis revealed an increased activity in limbic areas such as the parahippocampal gyrus and the thalamus, but also in sub-lobar areas such as the lentiform nucleus (putamen and lateral globus pallidus). In line with our results, there is evidence about the involvement of the parahippocampal gyrus in complex emotional processes, in particular for processing negative (see the review by Lew and Semendeferi, 2017) and uncertain stimuli (M. Zhang et al., 2016). Lesions in this area may lead to a decrement in sensitivity towards negative stimuli such as unpleasant music (Gosselin et al., 2006). Also, functional abnormalities in BD have been found in fronto-limbic structures involved in impulsivity, mood regulation, and emotion processing, including the hippocampal and parahippocampal cortex (Almeida et al., 2009; Delvecchio et al., 2013; Fleck et al., 2011; Houenou et al., 2011; Price and Drevets, 2012; Teng et al., 2014). Functional alterations in hippocampal-parahippocampal areas have been widely reported by previous meta-analyses on emotion processing in SCZ (Delvecchio et al., 2013; Dong et al., 2018; H. J. Li et al., 2010; Taylor et al., 2012). Interestingly, parahippocampal/hippocampal functional anomalies have been reported also in individuals at risk of psychosis during affective tasks (Bourque et al., 2017; O'Brien et al., 2020), and the reduced thickness of parahippocampal gyri has been identified as associated with genetic risk of both BD and SCZ (Hulshoff Pol et al., 2012).

Concerning the right thalamus, a meta-analysis on face emotion processing in healthy individuals reported its activation during disgusted faces processing when contrasted with neutral faces (Fusar-Poli et al., 2009). Furthermore, thalamic lesions are associated with impairments in facial affect recognition and poorer performance in sadness identification (Cheung et al., 2006). Higher activity in the thalamus may then represent heightened sensorial processing of visual negative stimuli (Delvecchio et al., 2012). Indeed, this area is involved in information processing (Castro-Alamancos, 2004; Pessoa, 2017) and it is more active during emotional scene processing when contrasted to neutral images (Sabatinelli et al., 2011). Thalamic morphometric alterations have been reported in both BD I patients not treated with lithium (Radenbach et al., 2010) and SCZ patients (Haijma et al., 2013; Wright et al., 2000). Further, even when directly compared, SCZ and BD revealed shared structural abnormalities in the thalamus (Dasari et al., 1999; Lee et al., 2020; McIntosh et al., 2004), confirming its involvement in both pathologies. Interestingly, antipsychotic drugs - that are often taken in both disorders - seem to act on the cellular activation in the thalamus (Cohen et al., 1998, 2003), and increase the volume of this area (Dazzan et al., 2005; Gur et al., 1998).

Both the thalamus and basal ganglia play an important role in the reward circuit (Haber and Knutson, 2010; Lapomarda et al., 2021a, 2021b) In addition, basal ganglia are involved in affective processes (Péron et al., 2017). In fact, alterations in this region are associated with abnormal global face emotion recognition and with difficulties in the identification of fearful, disgusted, and angry faces (Cheung et al., 2006). Moreover, the basal ganglia calcification can be associated with pathological mood alterations and psychosis (Johnson et al., 2013). Accordingly, evidence exists about structural anomalies of basal ganglia at early stage of BD (Strakowski et al., 2005), as well as shape alterations in BD (Hwang et al., 2006). Similarly, SCZ patients show altered basal ganglia volume and shape (Hirjak et al., 2015; Mamah et al., 2007; van Erp et al., 2016). These alterations seem to play a role in impulsivity as well, when considering dysfunctions in the interplay of cortical-limbic structures (e.g. anterior cingulate cortex, basal ganglia, insula) (Brown et al., 2006; Lapomarda et al., 2021a, 2021b). Finally, dysregulation of dopaminergic neurons in the basal ganglia (Haber, 2014) seems to cause an excessive salience attribution to neutral stimuli (see the review by Howes and Kapur, 2009), playing a crucial role in the psychotic manifestations of both disorders (for reviews see Kapur, 2003; Seeman and Kapur, 2000; Strakowski, 2014; Toda and Abi-Dargham, 2007; Walderhaug et al., 2011).

To sum up, we found that during the visual processing of negative valenced emotional stimuli both SCZ and BD patients show hyperactivation in the parahippocampal area, which is involved in the processing of uncertain (M. Zhang et al., 2016) and negative stimuli (see Lew and Semendeferi, 2017), as well as in basal ganglia and thalamus, which are usually implied in mood, emotional process and reward processing (Buhle et al., 2014; Haber and Knutson, 2010; Hsu et al., 2014; Johnson et al., 2013). As a matter of fact, abnormalities in this circuit have been linked to emotional disturbances (Delvecchio et al., 2012), as parahippocampal lesions can lead to decreased sensitivity towards unpleasant stimuli, perceiving them as pleasant (Gosselin et al., 2006) and thalamic and basal ganglia hyperactivation seem to amplify the processing of emotionally salient stimuli (Delvecchio et al., 2012). Abnormal salience attribution has been previously related to psychosis (for a review see Kapur, 2003). Taken together, our conjunction analysis data suggest that both BD and SCZ patients are characterised by an altered processing of negative emotional stimuli that leads them to elaborate negative cues as more unpleasant and intense than healthy participants.

### 4.2 Differences between schizophrenia and bipolar disorder: schizophrenia functional abnormalities

Despite the commonalities, our data also suggest some specificities for each disorder. SCZ patients display higher activations in subcortical areas, such as the amygdala, hippocampus and parahippocampal area, uncus and claustrum when compared to both HC and BD. Previous studies suggest the involvement of the amygdala in several aspects of negative emotions (such as emotional learning, response, recognition), social cognition, and social behaviour (reviewed in Schumann et al., 2011). Beside the well-known association with fear and anxiety (Fox and Shackman, 2019; Grecucci et al., 2020a; Sah, 2017; Saviola et al., 2020), the amygdala has been associated with other negative emotions, such as shame (Grecucci et al., 2021; Piretti et al., 2020), anger perception (Sorella et al., 2021), and sad or fearful facial expressions processing (Fusar-Poli et al., 2009). Alterations in the amigdalo-hippocampal complex have been reported to be associated with schizophrenia neurodevelopment and emotional dysfunctions (Aleman and Kahn, 2005; Bolton et al., 2012; Ganzola et al., 2014; Taylor et al., 2012).

Our meta-analysis shows that claustrum may play a role in the symptomatology of the SCZ. In a review, Crick and Koch (2005) speculated that the claustrum might be involved in consciousness (see also Koubeissi et al., 2014). Claustral white matter alterations in SCZ patients have been associated with symptoms unawareness (Antonius et al., 2011). Furthermore, claustral structural abnormalities have been detected in SCZ (Bernstein et al., 2016), in particular in patients with more severe delusional symptoms (Cascella et al., 2011). This area seems also to play a role in hallucinations during the administration of hallucinatory drugs or in Parkinson disease (for a review see Smith et al., 2020), and claustral white matter excess has been found in SCZ patients with hallucinations when compared with the ones that do not present this symptom (Shapleske et al., 2002). Also, a meta-analytic study reported that the claustrum activates during auditory verbal hallucinations (van Lutterveld et al., 2013). Interestingly, claustral functional hyperactivations have also been reported in SCZ patients with severe negative symptoms (Galeno et al., 2004).

Moreover, hyperactivation of the left superior temporal gyrus and the left fusiform gyrus seems to distinguish SCZ from BD. Beside their relevance for emotions (Vytal and Hamann, 2010), the left superior temporal gyrus has been linked to severe auditory verbal hallucinations in SCZ (Modinos et al., 2013). Similarly, some data suggest the involvement of the uncus in hallucinations (Fortuna et al., 2001; Roberts et al., 2001). The left parahippocampal area is involved in hallucinations as well, as its deactivation seems to predict auditory verbal hallucinations in SCZ (Diederen et al., 2010).

In sum, these results highlight the presence of specific increased activations in SCZ in regions that are not limited to the emotional domain (Bora and Pantelis, 2016; Goghari and Sponheim, 2013; Yalcin-Siedentopf et al., 2014), but that are also involved in other cognitive domains and psychotic manifestations proper to the disorder (for a review see Waters et al., 2012; Sorella et al., 2019).

### 4.3 Differences between schizophrenia and bipolar disorder: bipolar disorder functional abnormalities

Differently from SCZ, activation abnormalities in BD were mainly localised in the medial and dorsolateral prefrontal cortex (Brodmann, 1909, cited in Catani, 2019). These areas are involved in emotional appraisal (Ninivaggi, 2020), emotion regulation (Grecucci et al., 2019; Grecucci et al., 2020b; Hou et al., 2017; Messina et al., 2015; Pappaianni et al., 2020; Pozzi et al., 2021; Sulpizio et al., 2021), and response inhibition (Aron et al., 2003; Chikazoe et al., 2007; Jacobson et al., 2011).

Abnormal activation in the right inferior frontal gyrus (IFG) and in the right middle frontal gyrus (MFG) in BD patients has been previously reported in a meta-analysis on response inhibition (Hajek et al., 2013; see also Stefanopoulou et al., 2009). Interestingly, abnormal response inhibition has been proposed to be the most relevant cognitive endophenotype of bipolar disorder (Bora et al., 2009; Lapomarda et al., 2021a, 2021b).

Several data support the role of IFG, MFG and precentral gyrus in emotion regulation in healthy population (Brandl et al., 2019; Frank et al., 2014; Grecucci et al., 2013; Kohn et al., 2014; Morawetz et al., 2017). Emotion regulation aberrations associated to frontal anomalies have been frequently reported in BD (Kanske et al., 2015; Khafif et al., 2021; Sankar et al., 2021; L. L. Zhang et al., 2018). In their review, Kurtz et al. (2021) identified emotion regulation deficits as a potential marker of BD risk.

### 4.4 General discussion

The present study aimed to better characterise similarities and differences between SCZ and BD in the affective dimension of the *expanded continuum hypothesis* conceptualised by Sorella et al. (2019). By analysing studies about the visual perception of negative stimuli, we were able to point out shared abnormalities in a thalamic-parahippocampal-basal ganglia circuit. According to the above-mentioned literature, this circuit may be responsible for the greater sensitivity, heightened sensorial perception, and excessive salience attribution towards emotional stimuli proper to the two disorders. This result supports the hypothesis of shared abnormalities between 10.13039/100011025SCZ and 10.13039/100017412BD in the affective domain, specifically that of emotion perception, providing further evidence about the existence of an *expanded continuum* (Sorella et al., 2019).

Nevertheless, this study also highlighted differences between patients. On one hand, SCZ patients show specific functional aberrations in areas possibly involved in emotional processing, emotional memory, and hallucinations. On the other hand, BD patients show abnormalities in regions known to be crucial for emotion regulation and response inhibition. When considering affective and emotional brain regions mainly involved in perceptual, psychotic and memory processes, our data seem to support a greater impairment in 10.13039/100011025SCZ. On the other hand, when considering affective brain regions mainly involved in emotion regulation and response inhibition, our analysis shows stronger difficulties of BD patients. These results further support the *expanded continuum hypothesis* (Sorella et al., 2019), sustaining the presence of more severe affective dysfunctions in 10.13039/100017412BD, but framing them in the domain of emotion regulation.

## 5 Limitations

Our results should be considered in the light of some limitations. Starting from the selection criteria, the choice of including studies that considered emotional stimuli in contrast to their neutral counterparts (neutral faces and neutral IAPS) led us to exclude several studies that took into account different modalities (e.g., fixation cross, blank screen, shapes, scrambled or erased faces) as control condition. Nevertheless, this conservative criterion allowed us to reduce the presence of noise in the data. Another limitation is that the use of neutral faces as control condition has been questioned in light of the fact that these stimuli seem to be abnormally processed and misperceived by both schizophrenia and bipolar disorder patients when compared to healthy controls (Filkowski and Haas, 2017): instead of being perceived as a non-emotional baseline, neutral faces seem to be elaborated as fearful/angry by schizophrenics, while bipolars seem to process them as sad, showing a “negativity bias” towards neutral faces (reviewed by Filkowski and Haas, 2017).

Furthermore, previous studies suggested that BD I and BD II patients mildly differ in emotion perception (Derntl et al., 2009; B. B. Zhang et al., 2018) and emotion regulation (Caseras et al., 2015) from both behavioural and neural point of view. Unfortunately, our meta-analysis included only few BPD II patients due to the lack of studies focusing on this category of patients. In addition, in our selection criteria and analyses we did not consider the mood state nor the symptomatology of both patients’ groups. According to the emotional-response model elaborated by Bigot et al., in 2020, BD mood states play a crucial role in emotional processing, in particular in valence and intensity attribution. Also, the remitted and symptomatic states of SCZ patients seem to have consequences on emotion recognition (Maat et al., 2015).

To conclude, even though the present study results are novel and relevant, our meta-analysis did not include studies on emotion regulation: further studies on this topic are necessary to deepen it and test further the *continuum hypothesis*.

## 6 Conclusion

The present study aimed to shed light on the affective dimension of the *expanded continuum* hypothesised by Sorella et al. (2019) between schizophrenia (SCZ) and bipolar disorder (BD). To address this issue, we applied a coordinate-based meta-analytic approach to neuroimaging data. Our results highlight the presence of shared abnormal right-sided circuit in limbic and sub-lobar areas for both classes of patients. This data provides evidence for common alterations between SCZ and BD during the visual processing of negative stimuli. Nevertheless, the presence of distinct functional brain abnormalities – mainly located in the brainstem, limbic, temporal, and sub-lobar areas in SCZ, and in right frontal areas in BD – suggests that these pathological conditions diverge in some aspects of emotion perception. To conclude, our results provided new fresh knowledge on the affective dimension of the *expanded continuum hypothesis*. Future studies are needed to further understand the similarities and differences between these two complex disorders, in light of the affective, psychotic and cognitive dimensions of the *continuum*. Such findings may help better characterise similarities and differences between BD and SCZ for what concerns the affective side of the *continuum hypothesis*, possibly improving future diagnostic evaluations and personalised treatments of these disorders.

## Open practices statement

The meta-analysis data and materials are available at https://osf.io/cya9h/?view_only=0985efa9eca448b5a417a582c1b39c3d. The experiment was not preregistered.

## Funding

This research did not receive any fund, grant or other support from funding agencies in the public, commercial, or not-for-profit sectors.

## Authors’ contribution

**AG, CO, IM**: study design, analyses, figures, paper writing. **GL, SS**, paper writing, editing of the final version.

## Declaration of competing interest

The authors declare that they have no known competing financial interests or personal relationships that could have appeared to influence the work reported in this paper.

## Data Availability

Analized data can be found following the link reported in the section "Open practices statement". The MRI coordinates used can be found in the papers reported in Table 1
