# Supplementary material for: Perceiving visual negative stimuli in schizophrenia and bipolar disorder: Meta-analytic evidence of a common altered thalamic-parahippocampal-basal ganglia circuit
Source: Neuroimage Rep. 2023 May 8;3(2):100173. doi: 10.1016/j.ynirp.2023.100173 (PMC12172923; doi:10.1016/j.ynirp.2023.100173)
Supplement: Multimedia component 1 [file mmc1.docx]

**SUPPLEMENTARY MATERIALS**

| N° paper | Studies | Medication | Sex | Task description |
| --- | --- | --- | --- | --- |
| SCHIZOPHRENIA | | | | |
| 1 | Hall et al., 2008 | 16 atypical antipsychotic  3 antipsychotic | 12M, 7F (SCZ) 16M, 8F (HC) | Select the gender of the face by pressing a button. |
| 2 | H. J. Li et al., 2012 | NA number (chlorpromazine equivalents for the whole SCZ sample are given) | 6M, 6F (SCZ) 6M, 6F (HC) | Judge whether the facial expressions were positive or negative, and pressed one of two buttons accordingly. |
| 3 | Michalopoulou et al., 2008 | 8 conventional antipsychotic 3 atypical antipsychotic | 9M, 2F (SCZ) 5M, 4F (HC) | Indicate the gender of the face by moving a joystick. |
| 4 | Mier et al., 2014 | 100% antipsychotic 37% antidepressants 27% anticonvulsants | 64%M, 36%F (SCZ) 68%M, 32%F (HC) | Identify the emotion as fast as possible by pressing a button. |
| 5 |  | 100% antipsychotic 37% antidepressants 27% anticonvulsants | 64%M, 36%F (SCZ) 68%M, 32%F (HC) | Identify the emotion as fast as possible by pressing a button. |
| 6 |  | 100% antipsychotic 37% antidepressants 27% anticonvulsants | 64%M, 36%F (SCZ) 68%M, 32%F (HC) | Identify the emotion as fast as possible by pressing a button. |
| 7 | Williams et al., 2004 | 14 risperidone 7 olanzapine 5 clozapine 1 quetiapine | 17M, 10F (SCZ paranoid + non-paranoid) 14M, 8F (HC) | Select the gender of the face by pressing a button. |
| 8 | Williams et al., 2007 | PARANOID + NON-PARANOID INFORMATION 13 risperidone 7 olanzapine 6 clozapine 1 quetiapine  1 (paranoid) anticholinergic medication | 8M, 5F (SCZ paranoid) 17M, 10F (HC)* | Select the gender of the face by pressing a button. |
| 9 |  | PARANOID + NON-PARANOID INFORMATION 13 risperidone 7 olanzapine 6 clozapine 1 quetiapine | 9M, 5F (SCZ non-paranoid) 17M, 10F (HC)* | Select the gender of the face by pressing a button. |
| 10 |  | PARANOID + NON-PARANOID INFORMATION 13 risperidone 7 olanzapine 6 clozapine 1 quetiapine  1 (paranoid) anticholinergic medication | 8M, 5F (SCZ paranoid) 17M, 10F (HC)* | Select the gender of the face by pressing a button. |
| 11 |  | PARANOID + NON-PARANOID INFORMATION 13 risperidone 7 olanzapine 6 clozapine 1 quetiapine | 9M, 5F (SCZ non-paranoid) 17M, 10F (HC)* | Select the gender of the face by pressing a button. |
| 12 |  | PARANOID + NON-PARANOID INFORMATION 13 risperidone 7 olanzapine 6 clozapine 1 quetiapine  1 (paranoid) anticholinergic medication | 8M, 5F (SCZ paranoid) 17M, 10F (HC)* | Select the gender of the face by pressing a button. |
| 13 |  | PARANOID + NON-PARANOID INFORMATION 13 risperidone 7 olanzapine 6 clozapine 1 quetiapine | 9M, 5F (SCZ non-paranoid) 17M, 10F (HC)* | Select the gender of the face by pressing a button. |
| 14 |  | PARANOID + NON-PARANOID INFORMATION 13 risperidone 7 olanzapine 6 clozapine 1 quetiapine  1 (paranoid) anticholinergic medication | 8M, 5F (SCZ paranoid) 17M, 10F (HC)* | Select the gender of the face by pressing a button. |
| 15 |  | PARANOID + NON-PARANOID INFORMATION 13 risperidone 7 olanzapine 6 clozapine 1 quetiapine | 9M, 5F (SCZ non-paranoid) 17M, 10F (HC)* | Select the gender of the face by pressing a button. |
| 16 |  | PARANOID + NON-PARANOID INFORMATION 13 risperidone 7 olanzapine 6 clozapine 1 quetiapine  1 (paranoid) anticholinergic medication | 8M, 5F (SCZ paranoid) 17M, 10F (HC)* | Select the gender of the face by pressing a button. |
| 17 |  | PARANOID + NON-PARANOID INFORMATION 13 risperidone 7 olanzapine 6 clozapine 1 quetiapine | 9M, 5F (SCZ non-paranoid) 17M, 10F (HC)* | Select the gender of the face by pressing a button. |
| 18 |  | PARANOID + NON-PARANOID INFORMATION 13 risperidone 7 olanzapine 6 clozapine 1 quetiapine  1 (paranoid) anticholinergic medication | 8M, 5F (SCZ paranoid) 17M, 10F (HC)* | Select the gender of the face by pressing a button. |
| 19 |  | PARANOID + NON-PARANOID INFORMATION 13 risperidone 7 olanzapine 6 clozapine 1 quetiapine | 9M, 5F (SCZ non-paranoid) 17M, 10F (HC)* | Select the gender of the face by pressing a button. |
| 20 | Surguladze et al., 2011 | 12 flupentixol decanoate 2 fluphenazine decanoate 1 haloperidol decanoate 1 pipothiazine palmitate | 10M, 6F (SCZ conventional antipsychotic) 8M, 8F (HC) | Select the gender of the face by pressing a button. |
| 21 |  | 16 risperidone long-acting injections | 7M, 9F (SCZ risperidone) 8M, 8F (HC) | Select the gender of the face by pressing a button. |
| 22 | Lindner et al., 2014 | 36 second generation antipsychotics 14 antidepressant 4 anticonvulsives 3 additional typical antipsychotics 1 lithium | 22M, 14F (SCZ) 27M, 13F (HC) | Passive view: attentively watch and memorise the facial stimuli. |
| 23 |  | 36 second generation antipsychotics 14 antidepressant 4 anticonvulsives 3 additional typical antipsychotics 1 lithium | 22M, 14F (SCZ) 27M, 13F (HC) | Passive view: attentively watch and memorise the facial stimuli. |
| 24 | Rauch et al., 2010 | 5 one atypical neuroleptic 4 two atypical neuroleptics 3 combination of atypical and typical medication | 9M, 3F (SCZ) 7M, 5F (HC) | Rate if the masked face expresses a rather negative or rather positive emotion by pressing a button. |
| 25 | Lindner et al., 2016 | 36 second generation antipsychotics 14 antidepressant 3 anticonvulsives 3 additional typical antipsychotics 1 lithium | 23M, 13F (SCZ with flat affect + without) 27M, 13F (HC) | View: attentively watch and memorise the facial stimuli. |
| 26 |  | 36 second generation antipsychotics 14 antidepressant 3 anticonvulsives 3 additional typical antipsychotics 1 lithium | 23M, 13F (SCZ with flat affect + without) 27M, 13F (HC) | View: attentively watch and memorise the facial stimuli. |
| 27 |  | 36 second generation antipsychotics 14 antidepressant 3 anticonvulsives 3 additional typical antipsychotics 1 lithium | 23M, 13F (SCZ with flat affect + without) 27M, 13F (HC) | View: attentively watch and memorise the facial stimuli. |
| 28 |  | 36 second generation antipsychotics 14 antidepressant 3 anticonvulsives 3 additional typical antipsychotics 1 lithium | 23M, 13F (SCZ with flat affect + without) 27M, 13F (HC) | View: attentively watch and memorise the facial stimuli. |
| 29 | Das et al., 2007 | 5 no medication 4 olanzapine 3 risperidone 1 quetiapine 1 amisulpiride | 14M (SCZ first episode SCZ) 14M (HC) | Actively attend to the facial stimuli in order to attend a post-scan briefing regarding these stimuli. |
| 30 |  | ** 5 no medication 4 olanzapine 3 risperidone 1 quetiapine 1 amisulpiride | 13M (SCZ first episode SCZ) 14M (HC) | Actively attend to the facial stimuli in order to attend a post-scan briefing regarding these stimuli. |
| 31 | Becerril & Barch, 2011 | 92% atypical medications only 2.6% combination of typical/atypical | 66%M, 34%F (SCZ) 66%M, 34%F (HC) | Press the target button if the stimulus on the screen is the same as the one seen 2 trials prior; otherwise, press the nontarget button. |
| 32 | Reske et al., 2009 | 9 haloperidol 9 risperidone | 10M, 8F (first episode SCZ) 10M, 8F (HC) | Press the left or the right button if the facial stimulus is better described by the word in the left or in the right position. |
| 33 | Takahashi et al., 2004 | 11 atypical neuroleptics | 10M, 5F (SCZ) 9M, 6F (HC) | Indicate how each picture made the participants feel, categorizing their emotions in three categories (neutral, unpleasant and pleasant). |
| 34 | Mendrek et al., 2012 | ***22 at least one atypical antipsychotic (8 clozapine 8 risperidone 6 olanzapine 3 quetiapine 1 ziprasidone) | 17F (SCZ follicular phase) 15F (HC) | Indicate if the image contains a person/part of it by pressing a button. |
| 35 |  | ***22 at least one atypical antipsychotic (8 clozapine 8 risperidone 6 olanzapine 3 quetiapine 1 ziprasidone) | 17F (SCZ luteal phase) 15F (HC) | Indicate if the image contains a person/part of it by pressing a button. |
| 36 | Champagne et al., 2012 | 43 at least one atypical antipsychotic (28 one, 15 two) (20 clozapine 15 risperidone 12 olanzapine 9 quetiapine 2 ziprasidone) | 22M, 21F (SCZ) 22M, 21F (HC) | Passive view the stimuli. |
| 37 | Hägele et al., 2016 | 1 lorazepam 18 antipsychotics | 23M, 14F (SCZ) 22M, 18F (HC) | Passive view and confirm picture view by pressing a button with the right thumb. |
| 38 | Lakis et al., 2011 | 27 one antipsychotic 9 two antipsychotics 1 three antipsychotics (19 clozapine 12 olanzapine 11 risperidone 7 quetiapine) | 19M, 18F (SCZ) 19M, 18F (HC) | Determine if the image is old (previously presented) or new by pressing the correct button. |
| 39 |  | 27 one antipsychotic 9 two antipsychotics 1 three antipsychotics (19 clozapine 12 olanzapine 11 risperidone 7 quetiapine) | 19M, 18F (SCZ) 19M, 18F (HC) | Determine if the image is old (previously presented) or new by pressing the correct button. |
| 40 | Anticevic et al., 2011 | ****28 stable medication for two weeks or more | ****78%M, 18F (SCZ) 74%M, 22%F (HC) | Indicate after the presentation of 1) a memoranda set of two shapes, 2) a delay period, 3) the presentation of distracters, and 4) a post-distracter delay, if the probe shown was present in the memoranda set or not. IAPS stimuli were used as distracters. |
| 41 | Diaz et al., 2011 | 11 antipsychotic 6 antidepressants 2 lithium | 10M, 1F (SCZ) 7M, 10F (HC) | Indicate after the presentation of 1) a memoranda segment consistent of words, and 2) a set of emotional or neutral IAPS distracters, which of the two presented words was shown during the memoranda phase.  Half of the participants had also to categorise facial and non-facial stimuli during the presentation of the distracters by pressing two different buttons. |
| BIPOLAR DISORDER | | | | |
| 1 | Lawrence et al., 2004 | 12 medication 6 combination treatments (9 mood stabilizers 5 selective serotonin reuptake inhibitors 5 atypical antipsychotics 4 sodium valproate 3 lithium 2 carbamazepine 1 lamotrigine) | c.60%M, c.40%F (BD-I euthymic) c.60%M, c.40%F (HC) | Select the gender of each face and press one of two buttons accordingly with the right thumb. |
| 2 |  | 12 medication 6 combination treatments (9 mood stabilizers 5 selective serotonin reuptake inhibitors 5 atypical antipsychotics 4 sodium valproate 3 lithium 2 carbamazepine 1 lamotrigine) | c.60%M, c.40%F (BD-I euthymic) c.60%M, c.40%F (HC) | Select the gender of each face and press one of two buttons accordingly with the right thumb. |
| 3 |  | 12 medication 6 combination treatments (9 mood stabilizers 5 selective serotonin reuptake inhibitors 5 atypical antipsychotics 4 sodium valproate 3 lithium 2 carbamazepine 1 lamotrigine) | c.60%M, c.40%F (BD-I euthymic) c.60%M, c.40%F (HC) | Select the gender of each face and press one of two buttons accordingly with the right thumb. |
| 4 |  | 12 medication 6 combination treatments (9 mood stabilizers 5 selective serotonin reuptake inhibitors 5 atypical antipsychotics 4 sodium valproate 3 lithium 2 carbamazepine 1 lamotrigine) | c.60%M, c.40%F (BD-I euthymic) c.60%M, c.40%F (HC) | Select the gender of each face and press one of two buttons accordingly with the right thumb. |
| 5 | Lennox et al., 2004 | 8 lithium carbonate 4 carbamazepine 4 olanzapine 3 sodium valproate 3 haloperidol 1 sulpiride | 8M, 2F (BD-I manic) 6M, 6F (HC) | Rate explicitly the intensity of the emotion (4 point scale) in the faces by the prompt ‘How happy ?’ in the happy faces condition or ‘How sad ?’ in the sad faces condition using a 4-button response box. |
| 6 | Malhi et al., 2007 | 7 mood-stabilising psychotropic medications (4 valproate, 1 of them + lamotrigine 3 lithium) | 10F (BD-I euthymic) 10F (HC) | Indentify the emotion expressed by the facial stimuli by pressing one of three reaction time buttons corresponding to the three expressions (neutral, disgust and fear). |
| 7 |  | 7 mood-stabilising psychotropic medications (4 valproate, 1 of them + lamotrigine 3 lithium) | 10F (BD-I euthymic) 10F (HC) | Indentify the emotion expressed by the facial stimuli by pressing one of three reaction time buttons corresponding to the three expressions (neutral, disgust and fear). |
| 8 | Jogia et al., 2008 | / | 3M, 5F (BD-I baseline) 5M, 7F (HC) | Indentify the emotion expressed by the facial stimuli by pressing the right (sad stimuli) or left (neutral stimuli) button. |
| 9 | Sagar et al., 2013 | 17 primarily mood stabiliser 11 antipsychotics 4 antidepressant 4 benzodiazepines 4 unmedicated | NA | Indicate the gender of the facial stimuli by pressing a button. |
| 10 | Marchand et al., 2011 | / | 16M (BD-II depressed) 19M (HC) | Identify if two identical faces with the same facial expression are displayed sequentially by pressing a button. |
| 11 | Surguladze et al., 2010 | 16 at least one psychotropic medication (9 lithium 7 other mood stabilizers 4 antipsychotic 2 antidepressant | 9M, 11F (BD-I euthymic) 10M, 10F (HC) | Indicate the gender of the facial stimuli by pressing a button with the right index or middle finger. |
| 12 | Mullin et al., 2012 | 16 mood stabilisers 12 antipsychotics 9 antidepressant 3 benzodiazepines | 8M, 14F (BD-I euthymic) 8M, 11F (HC) | Press the button if the stimulus presented is identical to the one shown two trials previously (facial expressions as distracters). |
| 13 | Chen et al., 2006 | 8 mood stabiliser and/or antidepressant (4 lithium carbonate 2 carbamazepine 2 sodium valproate 2 olanzapine 2 haloperidol) | 8M (BD-I manic) 2M, 6F (HC) | Rate the emotional intensity of the faces in response to the question “How fearful?” or “How sad?” appearing with the fearful or sad stimuli. + Rate the intensity of the colour of each face (“How red?”, or “How green?”, or “How blue?”). |
| 14 |  | 8 mood stabiliser and/or antidepressant (4 lithium carbonate 2 carbamazepine 2 sodium valproate 2 olanzapine 2 haloperidol) | 8M (BD-I manic) 2M, 6F (HC) | Rate the emotional intensity of the faces in response to the question “How fearful?” or “How sad?” appearing with the fearful or sad stimuli. + Rate the intensity of the colour of each face (“How red?”, or “How green?”, or “How blue?”). |
| 15 |  | 8 mood stabiliser and/or antidepressant (7 lithium carbonate 3 carbamazepine 2 sodium valproate 1 olanzapine 1 haloperidol 2 thyroxine 1 sulpiride 1 risperidone 1 stelazine 1 venlafaxine 1 amitriptyline) | 5M, 3F (BD-I depressed) 2M, 6F (HC) | Rate the emotional intensity of the faces in response to the question “How fearful?” or “How sad?” appearing with the fearful or sad stimuli. + Rate the intensity of the colour of each face (“How red?”, or “How green?”, or “How blue?”). |
| 16 |  | 8 mood stabiliser and/or antidepressant (7 lithium carbonate 3 carbamazepine 2 sodium valproate 1 olanzapine 1 haloperidol 2 thyroxine 1 sulpiride 1 risperidone 1 stelazine 1 venlafaxine 1 amitriptyline) | 5M, 3F (BD-I depressed) 2M, 6F (HC) | Rate the emotional intensity of the faces in response to the question “How fearful?” or “How sad?” appearing with the fearful or sad stimuli. + Rate the intensity of the colour of each face (“How red?”, or “How green?”, or “How blue?”). |
| 17 | Grotegerd et al., 2014 | 13 antidepressive medication (5 mirtazapin 3 venlafaxine 2 citalopram 2 escitalopram 1 tranylcypromine 1 duloxetin 1 nortriptyline) 15 antipsychotic medication (14 quetiapine 1 asenapine 1 olanzapine) 13 mood stabiliser (9 lithium 3 lamotrigine 3 valproric acid) | 11M, 11F (BD-I depressed) 11M, 11F (HC) | Rate if the masked facial stimuli (neutral faces) expressed positive or negative emotions by pressing one of four buttons. |
| 18 | Deveney et al., 2014 | 20 medicated (11 antidepressant 9 atypical antipsychotic 9 antiepileptic 2 lithium) | 7M, 15F (BD-I/II adults, euthymic or depressed) 7M, 12F (HC adults) | Rate how hostile the facial stimuli are by using a five-button response device. |
| 19 |  | 20 medicated (11 antidepressant 9 atypical antipsychotic 9 antiepileptic 2 lithium) | 7M, 15F (BD-I/II adults, euthymic or depressed) 7M, 12F (HC adults) | Rate how wide is the nose of the facial stimuli by using a five-button response device. |
| 20 | Rootes-Murdy et al., 2019 | 8 lithium (2 from the whole BD-I sample also assumed other mood stabilisers) | 2M, 6F (BD-I lithium responders, euthymic) 6M, 15F (HC) | Passive view the stimuli. |
| 21 |  | 4 lithium (2 from the whole BD-I sample also assumed other mood stabilisers) | 4F (BD-I lithium non-responders, euthymic) 6M, 15F (HC) | Passive view the stimuli. |
| 22 | Bermpohl et al., 2009 | 7 lithium 6 quetiapine 2 valproic acid 1 olanzapine 1 risperidone | 5M, 5F (BD-I manic) 5M, 5F (HC) | Passive view the stimuli and confirm the view of the picture by pressing a button. |
| 23 | Cerullo et al., 2014 | / | 32%M, 68%F (BD-I depressed) 33%M, 67%F (HC) | Press a button with the right index finger if targets (circles) are shown, otherwise press another button with the right middle finger. (IAPS stimuli used as distracters). |
| 24 | L. Li et al., 2019 | 13 lithium 13 antipsychotics 2 anticonvulsants 1 antidepressant | 3M, 10F (BD-I lithium, euthymic) 7M, 9F (HC) | Passive view the picture and subsequently rate the picture valence by pressing a button. |
| 25 |  | 16 valproate 16 antipsychotics 1 antidepressants 1 anxiolytics | 6M, 10F (BD-I valproate, euthymic) 7M, 9F (HC) | Passive view the picture and subsequently rate the picture valence by pressing a button. |
| 26 | Hägele et al., 2016 | 11 different medications (1 to 3 drugs including mood stabilizers, benzodiazepines and antipsychotics) | 5M, 7F (BD-I manic) 22M, 18F (HC) | Passive view and confirm picture view by pressing a button with the right thumb. |
| 27 | Ellard et al., 2019 | NA number (antidepressants, mood stabilisers, antipsychotics, anxiolytics) | 23M, 16F (BD-I depressed) 18M, 18F (HC) | Select the number that, in a set of three numbers, differs from the other ones by pressing a button. These numbers overlaid IAPS images. |

Supplementary Table S1: Main details and description of the studies included in the meta-analysis. * The article by Williams et al., 2007 reports in its Table 1, which contains demographical information, that the sex distribution (M/F) among the 13 HC is 17/10. ** In the masked condition of the experiment conducted by Das et al., 2007, one participant was excluded due to excessive movement, but we could not find information about the medication taken by this participant, so we reported details regarding the drugs assumed by the whole sample even in the unconscious condition. *** Demographical data are reported for the whole sample of the Mendrek et al., 2012 experiment, but some participants were lately excluded. **** The original sample of Anticevic et al., 2011 consisted of 28 SCZ and 24 HC, but 6 patients and 1 control were excluded after the scanning procedure. Nevertheless, the demographical data reported regard the whole sample, and so the ones in the present table.

| Cluster | Side | Label (Nearest Gray Matter within 5mm) | BA | Peaks (MNI) | | | ALE | *P* | *Z* |
| --- | --- | --- | --- | --- | --- | --- | --- | --- | --- |
|  |  |  |  | x | y | z |  |  |  |
| SCHIZOPHENIA HYPERACTIVATIONS (SCZ > HC) | | | | | | | | | |
| 1 | L | Limbic Lobe, Parahippocampal Gyrus, Amygdala |  | -30 | -4 | -22 | 0.029 | 2.244E-11 | 6.587 |
| 1 | L | Limbic Lobe, Parahippocampal Gyrus | 35 | -28 | -28 | -16 | 0.014 | 1.060E-5 | 4.252 |
| 1 | L | Temporal Lobe, Sub-Gyral, Hippocampus |  | -28 | -24 | -10 | 0.011 | 7.781E-5 | 3.782 |
| 1 | L | Sub-Lobar, Lentiform Nucleus, Lateral Globus Pallidus |  | -26 | -10 | -2 | 0.010 | 2.266E-4 | 3.507 |
| 1 | L | Limbic Lobe, Parahippocampal Gyrus | 20 | -40 | -32 | -20 | 0.009 | 5.108E-4 | 3.284 |
| 2 | R | Sub-Lobar, Lentiform Nucleus, Putamen |  | 30 | -12 | 4 | 0.010 | 1.882E-4 | 3.556 |
| 2 | R | Sub-Lobar, Lentiform Nucleus, Putamen |  | 32 | -20 | -10 | 0.010 | 2.182E-4 | 3.517 |
| 2 | R | Limbic Lobe, Parahippocampal Gyrus | 35 | 22 | -20 | -16 | 0.010 | 2.363E-4 | 3.496 |
| 2 | R | Sub-Lobar, Lentiform Nucleus, Putamen |  | 20 | 6 | 2 | 0.010 | 2.363E-4 | 3.496 |
| 2 | R | Sub-Lobar, Lentiform Nucleus, Putamen |  | 30 | 2 | -4 | 0.010 | 2.474E-4 | 3.484 |
| 2 | R | Limbic Lobe, Parahippocampal Gyrus, Hippocampus |  | 32 | -22 | -16 | 0.009 | 3.831E-4 | 3.365 |
| 2 | R | Sub-Lobar, Thalamus, Ventral Lateral Nucleus |  | 18 | -12 | 4 | 0.009 | 5.568E-4 | 3.260 |
| SCHIZOPHRENIA HYPOACTIVATION (SCZ < HC) | | | | | | | | | |
| 1 | L | Limbic Lobe, Parahippocampal Gyrus, Amygdala |  | -22 | -4 | -20 | 0.025 | 4.664E-9 | 5.743 |
| 1 | L | Limbic Lobe, Parahippocampal Gyrus | 35 | -22 | -28 | -22 | 0.015 | 1.553E-5 | 4.166 |
| 1 | L | Cerebellum, Anterior Lobe, Culmen |  | -14 | -32 | -16 | 0.009 | 5.927E-4 | 3.242 |
| 1 | L | Temporal Lobe, Sub-Gyral | 20 | -38 | -14 | -24 | 0.009 | 6.271E-4 | 3.226 |
| 1 | L | Brainstem, Midbrain, Red Nucleus |  | -6 | -26 | -14 | 0.009 | 8.721E-4 | 3.131 |

Supplementary Table S2: Schizophrenia brain activations compared to healthy controls.

| Cluster | Side | Label (Nearest Gray Matter within 5mm) | BA | Peaks (MNI) | | | ALE | *P* | *Z* |
| --- | --- | --- | --- | --- | --- | --- | --- | --- | --- |
|  |  |  |  | x | y | z |  |  |  |
| BIPOLAR HYPERACTIVATIONS (BD > HC) | | | | | | | | | |
| 1 | R | Limbic Lobe, Parahippocampal Gyrus | 28 | 22 | -16 | -28 | 0.016 | 3.921E-6 | 4.469 |
| 1 | R | Sub-lobar, Lentiform Nucleus, Putamen |  | 30 | 4 | -10 | 0.015 | 6.375E-6 | 4.364 |
| 1 | R | Sub-lobar, Lentiform Nucleus, Medial Globus Pallidus |  | 18 | 0 | -12 | 0.010 | 2.525E-4 | 3.478 |
| 1 | R | Limbic Lobe, Parahippocampal Gyrus, Amygdala |  | 32 | -8 | -22 | 0.009 | 4.620E-4 | 3.313 |
| 1 | R | Sub-lobar, Lentiform Nucleus, Medial Globus Pallidus |  | 14 | -2 | 0 | 0.008 | 9.756E-4 | 3.098 |
| 1 | R | Limbic Lobe, Parahippocampal Gyrus | 35 | 24 | -28 | -22 | 0.008 | 0.002 | 2.896 |
| BIPOLAR HYPOACTIVATION (BD < HC) | | | | | | | | | |
| 1 | R | Frontal Lobe, Middle Frontal Gyrus | 9 | 48 | 18 | 36 | 0.009 | 2.719E-4 | 3.458 |
| 1 | R | Frontal Lobe, Inferior Frontal Gyrus | 9 | 54 | 14 | 24 | 0.008 | 3.180E-4 | 3.416 |
| 1 | R | Frontal Lobe, Inferior Frontal Gyrus | 9 | 38 | 12 | 30 | 0.008 | 4.614E-4 | 3.313 |
| 1 | R | No Gray Matter found |  | 26 | 4 | 32 | 0.008 | 4.823E-4 | 3.301 |

Supplementary Table S3: Bipolar brain activations compared to healthy controls.
